# Epidemiology of acute kidney injury in intensive care units in Beijing: the multi-center BAKIT study

**DOI:** 10.1186/s12882-019-1660-z

**Published:** 2019-12-16

**Authors:** Li Jiang, Yibing Zhu, Xuying Luo, Ying Wen, Bin Du, Meiping Wang, Zhen Zhao, Yanyan Yin, Bo Zhu, Xiuming Xi, The Beijing Acute Kidney Injury Trial (BAKIT) workgroup, Yuan Xu, Jianxin Zhou, Ang Li, Jingyuan Liu, Wenxiong Li, Wenjin Chen, Jianguo Jia, Xi Zhu, Penglin Ma, Wei Chen, Dongxin Wang, Youzhong An, Qingyuan Zhan, Gang Li, Haitao Zhang, Bo Ning, Zhongjie He, Zhicheng Zhang, Yaxiong Sun, Shijie Jia, Yalin Liu, Rui Cheng, Qing Song, Jinning Liu, Yangong Chao, Huizhen Li, Li Feng, Ruochun Shi, Xiuming Xi, Li Jiang, Ying Wen, Bo Zhu, Meiping Wang, Qi Jiang, Peng Wang, Yujie Deng, Yan Sun, Yanyan Yin, Xin Zhang, Li Zhang, Zhen Zhao, Ying Wang, Ran Lou, Jing Wang

**Affiliations:** 1 Department of Critical Care Medicine, Fuxing Hospital, Capital Medical University, 20A Fuxingmenwai Street, Xicheng District, Beijing, 100038 China; 2 Medical Research and Biometrics Center, Fuwai Hospital, National Center for Cardiovascular Diseases, Peking Union Medical College, Chinese Academy of Medical Sciences, Room 101–106, Block A, Shilong West Road, Mentougou District, Beijing, 102300 China; 3 Department of Critical Care Medicine, Tiantan Hospital, Capital Medical University, 119 Nansihuanxi Road, Fengtai District, Beijing, 100070 China; 4 Departmrnt of General Practice, Beitaipingzhuang Community Health Service Center, Building 6, Wenhuiyuan, Wenhuiyuan South Road, Haidian District, Beijing, 100082 China; 5 Medical ICU, Peking Union Medical College Hospital, Peking Union Medical College and Chinese Academy of Medical Sciences, 1 Shuai Fu Yuan, Beijing, 100730 China; 6 Department of Epidemiology and Health Statistics, School of Public Health, Capital Medical University, 10 Xitoutiao, Youanmenwai, Fengtai District, Beijing, 100069 China; 7 Center of urology and metabolism, Beijing Rehabilitation Hospital, Capital Medical University, Xixiazhuang, Badachu Road, Shijingshan District, Beijing, 100114 China

**Keywords:** Acute kidney injury, Renal replacement therapy, Critical care medicine, Mortality, Epidemiology

## Abstract

**Background:**

Acute kidney injury (AKI) commonly occurs in intensive care units (ICUs), leading to adverse clinical outcomes and increasing costs. However, there are limited epidemiological data of AKI in the critically ill in Beijing, China.

**Methods:**

In this prospective cohort study in 30 ICUs, we screened the patients up to 10 days after ICU admission. Characteristics and outcomes were compared between AKI and non-AKI, renal replacement therapy (RRT) and non-RRT patients. Nomograms of logistic regression and Cox regression were performed to examine potential risk factors for AKI and mortality.

**Results:**

A total of 3107 patients were included in the final analysis. The incidence of AKI was 51.0%; stages 1 to 3 accounted for 23.1, 11.8, and 15.7%, respectively. The majority (87.6%) of patients with AKI developed AKI on the first 4 days after admission to the ICU. A total of 281 patients were treated with RRT. Continuous RRT with predilution, citrate for anticoagulation and femoral vein for vascular access was the most common RRT pattern (29.9%, 84 of 281). Patients with AKI were associated with longer ICU-LOS and higher mortality and costs (*P*<0.001). In patients treated with RRT, 78.6 and 28.5% of RRTs were dependent on the 7th and 28th days, respectively. The 28 day mortalities of non-AKI, AKI stages 1–3, and septic shock patients were 6.83, 15.04, 27.99, 45.18 and 36.5%, respectively.

**Conclusions:**

Approximately half of our ICU patients experienced AKI. The majority of patients with AKI developed AKI during the first 4 days after admission to the ICU. Continuous RRT with predilution, citrate for anticoagulation and femoral vein for vascular access was the most common RRT pattern in our ICUs. AKI was associated with a higher mortality and costs, incomplete kidney recovery and s series of adverse outcomes.

## Background

Acute kidney injury (AKI) is a life-threatening disease and global health burdens with increasing incidence in both developed and developing countries [1, 2]. AKI commonly occurs in the intensive care unit (ICU), and is caused by multiple risk factors, leading to adverse clinical outcomes, increasing costs, and the development of chronic kidney disease (CKD) [3–8]. The definition of AKI has evolved from the Risk, Injury, Failure, Loss, End-stage (RIFLE) criteria and the AKI Network (AKIN) classification to the Kidney Disease Improving Global Outcomes (KDIGO) classification [9–11]. A multinational epidemiological study using KDIGO criteria showed that the incidence of AKI in the ICU was 57.3% [12], which implies concern regarding AKI the in ICU globally with extremely high morbidity reported. It is essential for physicians, researchers, and health policy makers to establish an accurate incidence of AKI [12]. There have been were large epidemiological studies of patients hospitalized with AKI in the Chinese population [13, 14]. However, there have been limited epidemiological data on AKI in the ICU in mainland China reported [15]. Therefore we performed a cohort study of all the adult patients in 30 ICUs of 28 hospitals in Beijing to determine the incidence, risk factors, renal replacement therapy (RRT) practice, and the outcome of patients with AKI.

## Methods

### Study design

This is a multi-center prospective cohort study on the epidemiology of AKI in ICU patients in Beijing, China. A full list of the participating hospitals is provided in Additional file 1. The study was registered with the Chinese Clinical Trial Registry (ChiCTR-ONC-11001875). Thirty ICUs in 28 teaching hospitals (two of the hospitals include two ICUs, respectively) in Beijing participated in this prospective observational study between March 1, 2012, and August 31, 2012 (a 6-month period). The Ethics Committee of Capital Medical University, Fuxing Hospital and all other participating hospitals approved the informed consent waiver due to the anonymous and non-interventional nature of the study (2010FXHEC-KY026, Additional file 2). Patients admitted to any participating ICU during the study period were included. We excluded (1) patients under 18 years old, (2) undergoing any kind of RRT within 3 months, (3) kidney transplantation within 3 months, (4) anticipated length of stay in the ICU (ICU-LOS) for less than 24 h, and (5) readmission to the ICU during the study period.

### Definitions

AKI was defined by the KDIGO criteria [16]. Patients were categorized on the basis of serum creatinine and/or urine output; the criteria leading to the worst classification used. Baseline serum creatinine was defined as the lowest serum level during the preceding 3 months [17]. For patients without a baseline serum creatinine laboratory test result, the baseline was estimated by the modification of diet in renal disease (MDRD) equation and customized for the Chinese population, assuming a glomerular filtration rate (GFR) of 75 mL/min per 1.73 m^2^ [18]. Sepsis was defined as the combination of infection and systemic inflammatory response syndrome [19].

### Data collection

Data were recorded on the case-reported form (CRF) (Additional file 3). On admission, data regarding demographics, admission time point, in-hospital location before ICU admission, acute physiology age and chronic health evaluation II (APACHE II) score, simplified acute physiology score II (SAPS II) score, sequential organ failure assessment (SOFA) score, baseline serum creatinine, comorbidity, and medications administrated before ICU admission were collected. During the first 10 days after admission, daily vital signs, urine output per hour, daily fluid balance, serum creatinine, medications, interventions, possible causes for AKI, diagnosis and stage of AKI, detailed information of RRT including reasons for initiation of RRT, mode of RRT, and anticoagulant and dilution patterns were collected. Diagnosis of sepsis and association between sepsis and AKI were reported. Outcome measures were collected including mortality, ICU-LOS, costs, withholding or withdrawal of life-sustaining treatments (WH/WD), and RRT dependence on the 7th and 28th days.

### Statistical analyses

Continuous variables are presented as medians with interquartile ranges (IQRs), and compared by the Mann-Whitney U-test or Kruskal-Wallis ANOVA test. Categorical variables were compared using either the chi-square test or Fisher’s exact test when appropriate. Statistical descriptions and tests above were performed using SPSS version 17.0.1 (SPSS Inc., Chicago, IL, USA). Multivariable logistic regression with odds ratio (OR) and 95% confidence interval (CI) was performed to assess independent risk factors for AKI development. Cox proportional hazards regression analysis with hazard ratio (HR) and 95% CI was performed to examine whether the KDIGO stage was associated with mortality adjustment for baseline severity of illness, age and other factors. We used weighted estimators corresponding to each covariate derived from the fited logistic and Cox regression coefficients. The prognostic index was calculated by summing the number of risk points corresponding to each weighted covariate used to build the two nomograms. The specific codes used are provided in Additional file 4: Appendix 4. A *p* value of less than 0.05 was considered significant. The nomograms of logistic regression and Cox regression were performed using R 3.5.1. The function “lrm” of the package “rms” was used for the logistic regression. The function “cph” of the package “survival” was used for the Cox regression.

## Results

**Fig. 1:**
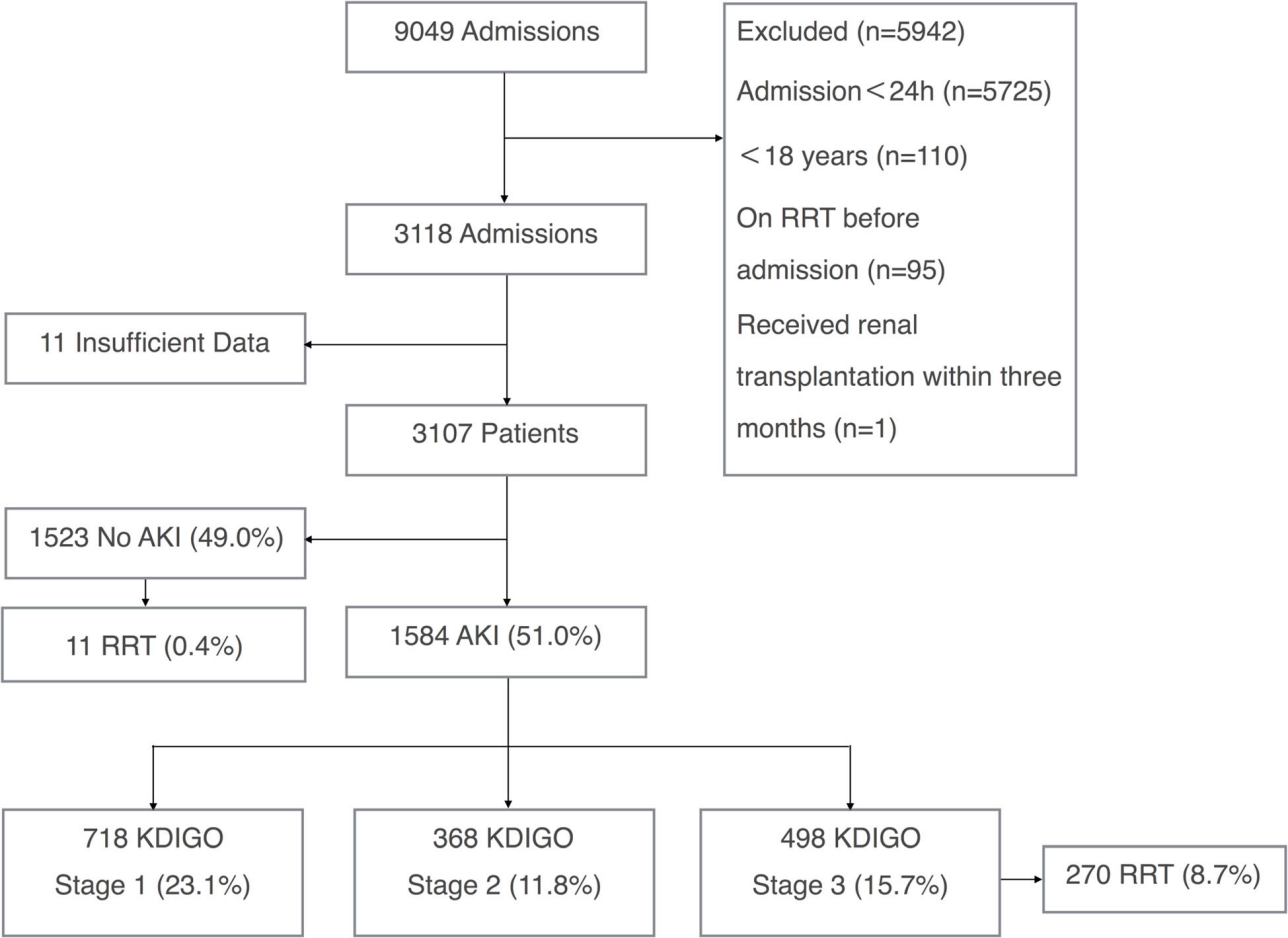
Flow chart. *AKI* acute kidney injury, *KDIGO* Kidney Disease: Improving Global Outcomes, *RRT* renal replacement therapy

A total of 3107 participants were included in the final analysis among the 9049 patients admitted to the participating ICUs during the study period. The flow chart is presented in Fig. 1. In comparison of the characteristics between patients with and without AKI, patients with AKI have a higher median age, baseline serum creatinine, APACHE II score, SAPS II score, SOFA score, non-renal SOFA score, more comorbidity, a higher rate of mechanical ventilation, use of inotropic agents and diuretics, and WH/WD. (Table 1).

**Table 1:** Characteristics and outcomes of included patients

	All patients	AKI	Non-AKI	*p*
Number of patients	3107 (100%)	1584 (50.9%)	1523 (49.1%)	
Age (years)	64 (51–77)	67 (53–78)	62(49–74)	< 0.001
Male	1912 (61.5%)	970 (61.2%)	942 (61.9%)	0.74
Baseline SCr	77 (60.2–93)	79 (63–94)	74 (58.8–89)	< 0.001
APACHE II score	14 (10–20)	17 (12–23)	12 (8–16)	< 0.001
SAPS II score	34 (26–45)	39 (30–52)	29 (23–38)	< 0.001
SOFA score	6 (3–8)	7 (4–10)	4 (2–7)	< 0.001
Non-renal SOFA score	5 (3–8)	6 (4–9)	4 (2–6)	< 0.001
Co-morbidity				
CHD	615 (19.8%)	370 (23.4%)	245 (16.1%)	< 0.001
CHF (NYHA IV)	217 (7.0%)	152 (9.6%)	65 (4.3%)	< 0.001
HT	1222 (39.3%)	687 (43.4%)	535 (35.1%)	< 0.001
DM	532 (17.1%)	320 (20.2%)	212 (13.9%)	< 0.001
COPD	166 (5.3%)	98 (6.2%)	68 (4.5%)	0.038
CKD	203 (6.5%)	167 (10.5%)	36 (2.4%)	< 0.001
CLF	91 (2.9%)	53 (3.3%)	38 (2.5%)	0.168
Sources of patients				
ED	774 (24.9%)	440 (27.8%)	334 (21.9%)	< 0.001
general wards	586 (18.9%)	383 (24.2%)	203 (13.3%)	< 0.001
post-operation	1627 (52.4%)	692 (43.7%)	935 (61.4%)	< 0.001
other ICUs	31 (1.0%)	21 (1.3%)	10 (0.7%)	< 0.001
other hospitals	89 (2.9%)	48 (3.0%)	41 (2.7%)	< 0.001
Medications before admission				
Aminoglycosides	47 (1.5%)	30 (1.9%)	17 (1.1%)	0.079
Glycopeptide antibiotics	50 (1.6%)	32 (2.0%)	18 (1.2%)	0.065
Radio-contrast media	560 (18%)	305 (19.3%)	255 (16.7%)	0.069
Mannitol	92 (3.0%)	45 (2.8%)	47 (3.1%)	0.751
NSAIDs	253 (8.1%)	152 (9.6%)	101 (6.6%)	0.003
ACEI/ARB	523 (16.8%)	281 (17.1%)	242 (15.9%)	0.179
Statin	352 (11.3%)	184 (11.6%)	168 (11.0%)	0.611
SCr	83 (62–116)	105.4 (72–161)	70.8 (56.7–89)	< 0.001
Sepsis	641 (20.6%)	459 (29.0%)	182 (12.0%)	< 0.001
Organ failure				
Respiratory failure	811 (26.1%)	538 (34.0%)	273(17.9%)	< 0.001
Acute hepatic failure	53 (1.7%)	37 (2.3%)	16 (1.1%)	0.006
Hematologic failure	44 (1.4%)	34 (2.1%)	10 (0.7%)	< 0.001
Shock	484(15.6%)	353(22.3%)	131 (8.6%)	< 0.001
Cardiogenic shock	117 (3.8%)	96 (6.1%)	21 (1.4%)	< 0.001
Hypovolemic shock	201 (6.5%)	130 (8.2%)	71 (4.7%)	0.004
Septic shock	222 (7.1%)	173 (10.9%)	49 (3.2%)	< 0.001
Obstructive shock	4 (0.1%)	2 (0.1%)	2 (0.1%)	< 0.001
MV	2344 (75.4%)	1260 (79.5%)	1084 (71.2%)	< 0.001
Vasopressors	930 (29.9%)	480 (30.3%)	450 (29.5%)	0.666
Inotropic agents	665 (21.4%)	366 (23.1%)	299 (19.6%)	0.02
Diuretics	1650 (53.1%)	1067 (67.4%)	583 (38.3%)	< 0.001
WH/WD	691 (22.2%)	398 (25.1%)	293 (19.2%)	< 0.001
ICU mortality	395 (12.7%)	346 (21.8%)	49 (3.2%)	< 0.001
28-day mortality	542 (17.4%)	438 (27.7%)	104 (6.8%)	< 0.001
ICU-LOS (days)	4(2–10)	5.5 (3–11)	3 (2–6)	< 0.001
ICU overall costs (RMB)	32,000 (17000–74,000)	42,000(21000–95,000)	26,000 (14000–51,000)	< 0.001
ICU daily costs (RMB)	6500 (4500–10,000)	6667 (4826–10,182)	6333 (4333–10,000)	< 0.001

Values are presented as n (proportion) or median (interquartile range)

*AKI* Acute kidney injury, *SCr* Serum creatinine, *CHD* Coronary heart disease, *CHF* Chronic heart failure, *NYHA* the New York heart association functional classification, *HT* Hypertension, *DM* Diabetes mellitus, *COPD* Chronic obstructive pulmonary disease, *CKD* Chronic kidney disease, *CLF* Chronic liver failure, *APACHE II* Acute physiology and chronic health evaluation II, *SAPS II* Simplified acute physiology score II, *SOFA* Sequential organ failure assessment score non-renal, *SOFA* Sequential organ failure assessment score without the renal component, *ED* Emergency department, *ICU* Intensive care unit, *NSAIDs* Non-steroidal antiinflammatory drugs, *ACEI* Angiotensin-converting enzyme inhibitor, *ARB* Angiotensin receptor blocker, *MV* Mechanical ventilation, *WH/WD* Withholding or withdrawal of life-sustaining therapy

### Incidence of AKI

**Fig. 2:**
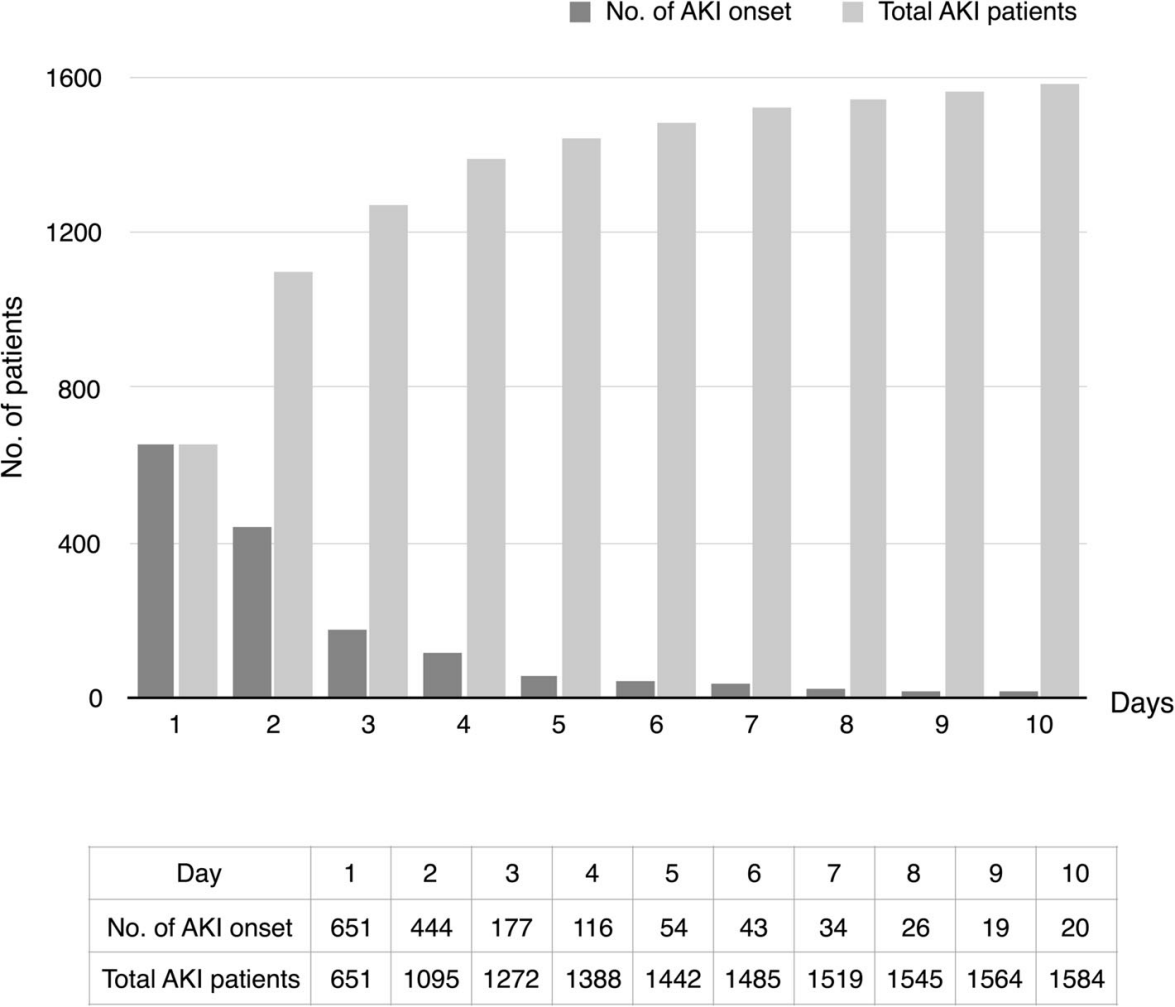
Daily AKI onset and accumulative AKI patients on the first 10 days. *No*. number, *AKI* acute kidney injury, *CRF* case report form, *KDIGO* the Kidney Disease: Improving Global Outcomes

The incidence of AKI was 51.0% (1584 of 3107) including stage 1 AKI 23.1% (718 of 3107), stage 2 AKI 11.8% (368 of 3107), and stage 3 AKI 15.7% (498 of 3107) (Fig. 1). The majority of patients with AKI (87.6%, 1388 of 1584) developed AKI during the first 4 days after admission to the ICU (Fig. 2).

### Causes and risk factors of AKI

**Fig. 3:**
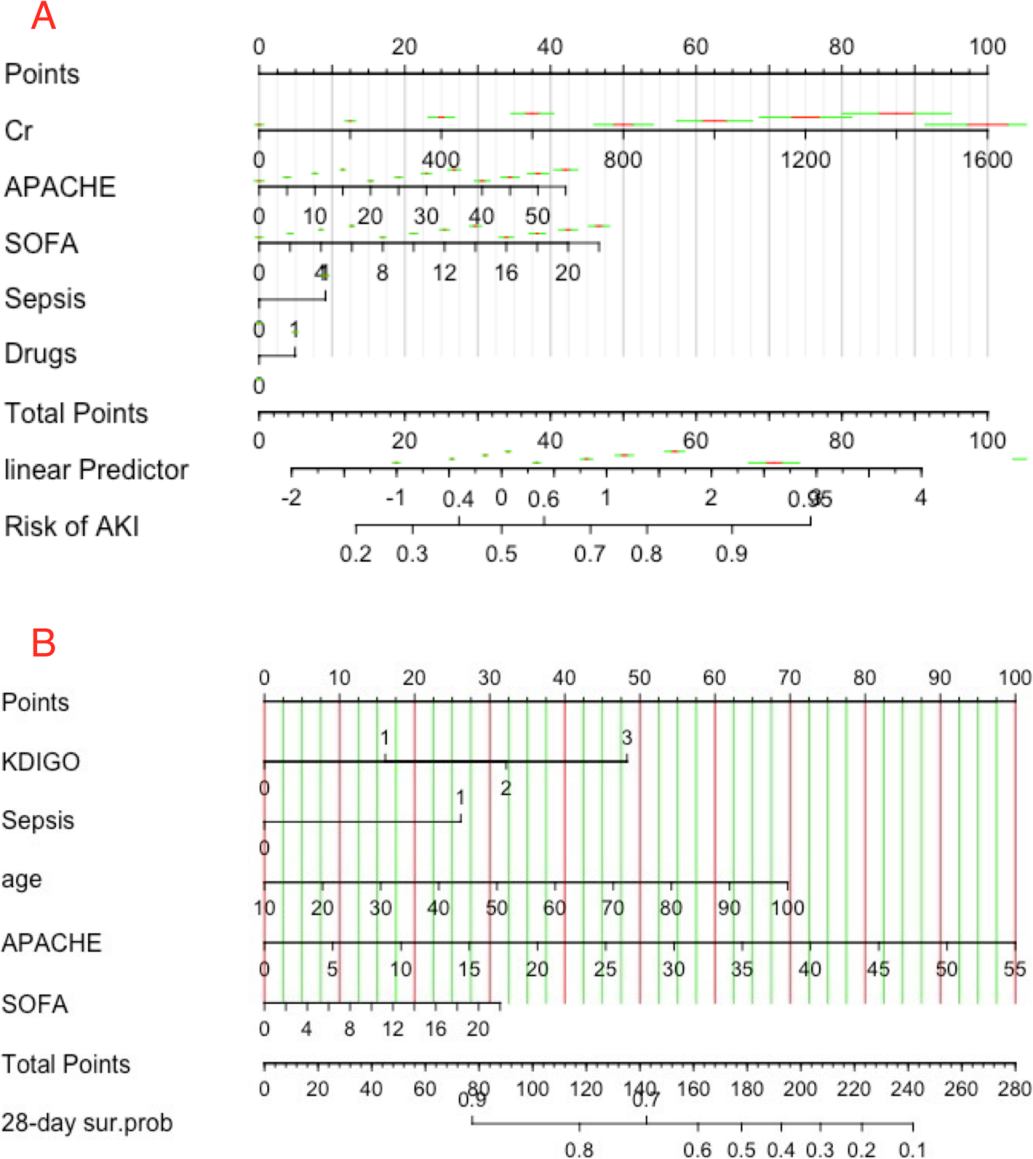
Nomogram of independent predictors for development of AKI and 28 day mortality. Each predictor with a given value can be mapped to the Points axis. The sum of these points can be referred to in the Total Points axis. Then the linear predictor and the probability of development of AKI (A) and survival (B) can be obtained from corresponding axis. The green bar indicates the 0.7 confidence limits for each score, and the short red bar corresponds to 0.1 confidence limits. *Cr* baseline creatinine, *APACHE* acute physiology and chronic health evaluation II, *SOFA* sequential organ failure assessment score, *Drugs* nephrotoxic drug exposure, KDIGO Kidney Disease: Improving Global Outcomes criteria and stage of AKI, *28-day sur.pro* probability of survival on the 28th day

Hypovolemia (25.4%), sepsis on ICU admission (22.2%) and low cardiac output (20.5%) were the top three possible causes. The logistic regression nomogram indicated that baseline creatinine (OR = 1.00; 95% CI 1.00–1.01), APACHE II score (OR = 1.05; 95% CI: 1.04–1.07), SOFA score (OR = 1.16; 95% CI 1.13–1.19), sepsis (OR = 1.88; 95% CI 1.56–2.27) and exposure to nephrotoxic drugs (OR = 1.41; 95% CI 1.19–1.66) might be independent predictors of AKI development (Fig. 3a). There were 876 patients diagnosed with sepsis on ICU admission and/or during ICU stays. Physicians reported that 296 (33.8%) cases of sepsis contributed to AKI, and 175 (20.0%) cases of sepsis were possibly associated with AKI development.

### Renal replacement therapy

A total of 281 patients were treated with RRT, including 270 patients with AKI (accounting for 17% of the AKI and 8.7% of all patients) and 11 non-AKI patients. The top four reasons to initiate RRT were anuria/oliguria (71.9%, 201/281), severe metabolic acidosis (25.6%, 72/281), hyperkalemia (21.7%, 61/281) and fluid overload (20.3%,57/281). Furthermore, 38.3% (109 of 281) of patients had multiple reasons for initiating RRT, with anuria/oliguria together with hyperkalemia being most commonly reported. The 11 non-AKI patients received RRT due to acute heart failure (*n* = 4, to reduce heart load), poisoning (*n* = 3, to detoxify), heat stroke (*n* = 2, for rapid hypothermia), and sever e sepsis (n = 2, to clear inflammatory media). The characteristics and outcomes of RRT patients are presented in Table 2. The majority of RRT procedures were continuous RRT (CRRT). Intermittent RRT (IRRT) was seldom used (2%). Continuous veno-venous hemofiltration (CVVH) was the most common mode. Among 277 patients with the modes of anticoagulant reported, sodium citrate (121, 43.7%) was the most commonly reported anticoagulation pattern, followed by no anticoagulation (68, 24.5%), unfractionated heparin (53, 19.1%) and low-molecular-weight heparin (22, 7.9%). For dilution patterns reported in 269 cases, predilution, combination of pre- and post-dilution, and postdilution accounted for 56.5, 25.3, and 18.2%, respectively. Of the 284 reported catheter insertion sites, the femoral vein was the most common (74.3%, 211/284; with 125 right femoral vein, 86 left femoral vein), followed by the internal jugular vein (23.6%, 67/284; with 48 right jugular vein, 19 left jugular vein), 5 subclavian vein and 1 arterio-venous fistula. Continuous RRT with predilution, citrate for anticoagulation and femoral vein for vascular access was the most common pattern (29.9%, 84 of 281). For adverse events, bleeding or oozing at the catheterization site was the most commonly reported (12.5%, 35 of 281), followed by gastrointestinal bleeding (9.6%, 27 of 281) and cutaneous bruise or ecchymoses (6.8%, 19 of 281). The 28-day mortality of patients treated with RRT was 48.8%. In 281 patients treated with RRT, 76.0% (173 of 220 survivors) of patients depended on RRT on the 7th day. Among 144 survivors on the 28th day, 41 patients (28.5%) were dependent on RRT; 25 (19.7%) patients depended on intermittent hemodialysis (IHD), and 16 (11.1%) depended on CRRT. The ICU-LOS, mortality rate and costs of RRT patients were significantly higher than those of non-RRT AKI patients (Table 2).

**Table 2:** Characteristics and outcomes of RRT patients

	RRT	Non-RRT	*P*
Number of patients	281	1314	
Characteristics			
Age	66 (52–79)	67 (53–78)	0.141
Male	178 (63.3%)	791 (60.2%)	<0.05
APACHE II	22 (17–29)	16 (11–22)	<0.05
ICU mortality	109 (38.8%)	236 (17.9%)	<0.05
28-day mortality	130 (46.3%)	303 (23.0%)	<0.05
ICU-LOS (days)	8 (5–17)	5 (3–10)	<0.05
ICU overall costs (RMB)	104,000 (60000–213,000)	37,000 (19000–87,000)	<0.05
ICU daily costs (RMB)	9765 (5580–14,625)	6143 (4333–9000)	<0.05
RRT dependent on 7th day	173/220 (78.6%)	NR	<0.05
RRT dependent on 28th day	41/144 (28.5%)	NR	<0.05

*APACHE II* Acute physiology and chronic health evaluation II, *ICU* Intensive care unit, *LOS* Length of stay, *NR* Not recorded, *RRT* Renal replacement therapy

### Clinical outcomes and costs

Patients with AKI had a significantly higher rate of withholding or withdrawing of life-sustaining treatments, ICU mortality, 28-day mortality, longer ICU-LOS, and higher ICU overall costs (Table 1). The 28-day mortality rate of non-AKI patients was 6.83%. The mortality rates of AKI stages 1–3 were 15.04, 27.99, and 45.18%, respectively. The 28-day mortality rate of the 917 septic AKI patients was 36.5%. The Cox regression nomogram indicated that a higher stage of AKI (HR = 1.35; 95% CI 1.25–1.47), higher age (HR = 1.01; 95% CI 1.01–1.02), high APACHE II (HR = 1.03; 95% CI 1.02–1.05) and SOFA scores (HR = 1.03; 95% CI 1.00–1.06), and sepsis (HR = 1.63; 95% CI1.35–1.98) were independent predictors of mortality (Fig. 3b).

## Discussion

Our results indicated a high incidence of AKI in the ICU. Approximately half of our ICU patients experienced AKI. The incidence in the present study was significantly higher than those in an international epidemiological study in 2005 [20] and the FINNAKI study [21], which might be attributed to the discrepancy in diagnostic criteria. A study comparing the three diagnostic criteria indicated that the KDIGO criteria identified more patients with AKI and were more predictive of short-term mortality [22]. The incidence in the present study was similar to the Acute Kidney Injury- Epidemiological Prospective Investigation (AKI-EPI) study [12]. The AKI-EPI study [12] was the first multinational epidemiological study in the ICU using the KDIGO criteria. Both AKI-EPI and our study demonstrated an unsatisfactory prevention and increasing burden of AKI. In our study, the top three possible causes reported by physicians were hypovolemia, sepsis on ICU admission and low cardiac output, which was similar to the result of the FINNAKI study (preceding AKI severe sepsis, pre-ICU hypovolemia and pre-ICU hypotension) [21]. Our logistic regression showed comprehensible risk factors. The RRT ratio in our study was similar to that of the FINNAKI study (8.6% vs 9.4%) [21], but lower than that of the AKI-EPI study (13.5%) [12]. The higher RRT ratio in the AKI-EPI study could be attributable to the higher ratio (KDIGO stage 3: 30.0%) of patients with more severe AKI in comparison with the other two studies (KDIGO stage 3: 15.7%; AKIN stage 3: 14.1%). The RRT pattern results indicated that CRRT was mainly chosen (97.9%) and that IRRT was seldom used. A previous observational study in French ICUs showed that 40% of RRT patients had CRRT and 60% had IRRT [23], which varied greatly from our results. Although the previous studies have shown no difference in clinical outcomes comparing CRRT and IRRT in the ICU [24–26], our results revealed a strong inclination to CRRT in our ICUs. The advantages of CRRT include better hemodynamic tolerance, accurate balance control and better clearance of the middle molecule [11, 27]. Besides our ICU physicians and nurses were familiar with CRRT and unfamiliar with IRRT. Our results showed that the femoral vein (74.6%) was the most common catheter location, differing from the recommendations in the guideline (the right internal jugular vein) [28]. Studies have suggested that the internal jugular vein might be preferable to the femoral vein to minimize dialysis catheter dysfunction and blood recirculation to improve RRT provision and reduce the risk of infection [29–32]. The reasons for our physicians preferring femoral access might be the advantages of convenience, efficiency and fewer complications in the operating procedure. The results indicated that citrate was the most commonly used for anticoagulant. Meta-analyses [33–35] suggested that citrate is preferable to heparin in anticoagulation for CRRT to prolong circuit life span, reduce the risk of bleeding [34, 35], and increase the delivered RRT dose [33]. Citrate is novel and has not been used for long in CRRT; nevertheless, our results indicated that it has already been widely used in Beijing. However, considering the advantages of better medical resources in Beijing, the capital city of China, the result may not be representative of the whole country. The distinction of guideline compliance with respect to vascular access and anticoagulation might imply that changes in medication are more acceptable for physicians than are changes in techniques. The mortality rate was comparable to the FINNAKI study [21]. The regression analysis indicated that a greater severity of AKI, and comorbidity of sepsis were associated with an increased risk of mortality, which was in accordance with the previous studies [12, 13, 21]. Our results showed a significantly higher ICU costs for RRT patients, which is reasonable and comparable to other studies [26, 35]. RRT patients had a higher AKI stage, more complications, higher disease severity score and longer ICU-LOS, all of which lead to higher costs [35]. Our results showed that increased AKI stage and septic AKI were associated with higher mortality, and those trends were also found in a study that evaluated patients with AKI with and without sepsis [36]. More than 1/4 of survivors were RRT dependent, which would lead to heavy burdens. There are strengths in our study. This is the first large, prospective, multicenter cohort study of AKI in the ICU in Beijing. We used the KDIGO criteria to evaluate the prevalence of AKI. Previous studies [22, 37, 38] indicated that the KDIGO criteria defined more patients with AKI in comparison with RIFLE and AKIN. Further analysis showed that the patients missed by RIFLE had higher mortality rate and longer hospital-LOS than the patients missed by KDIGO [22]. For the first time the incidence of AKI in the ICU for ten consecutive days was reported, to the best of our knowledge. The results revealed an obvious downtrend of AKI onset over time. We consider this result to be meaningful epidemiological data that might imply the necessity of key vigilance against the risk of AKI in the first 4 days after ICU admission. Furthermore, we investigated the comprehensive situation in RRT practice. Thus, we understood our insufficiencies and underlying causes, which are important areas for policy makers and physicians to make improvements. There were limitations in our study. First, our participating hospitals were all located in Beijing. As the capital city of China, Beijing enjoys better medical resources. Thus, the results of our study might not be representative of hospitals nationwide. Second, we used the MDRD equation to estimate the baseline serum creatinine for missing values, as recommended [18, 39]. However, the MDRD method may result in under- or overestimation of baseline creatinine [39, 40]. Third, we screened patients for AKI for the first 10 days after admission to the ICU. Thus, we were unable to analyze later-onset AKI. However, based on our results, the majority of patients with AKI (87.6%) had AKI onset in the first 4 days after admission. Our data on the occurrence of AKI during the first 10 days may imply that the onset of AKI in the ICU decreases over time, and new onset after 10 days would be minimal.

## Conclusions

There was a high incidence of AKI in the ICU. Approximately half of our ICU patients experienced AKI. The majority of patients with AKI developed AKI during the first four ICU days. For RRT patterns, continuous RRT, predilution, citrate, and femoral vein were the most commonly used RRT procedure, dilution mode, anticoagulant and vascular access, respectively. AKI was associated with increased mortality and costs, incomplete kidney recovery and a series of adverse outcomes. Higher AKI stage, septic-AKI and the need for RRT were associated with increased mortality.

## Supplementary information

**Additional file 1.** Full list of participating hospitals.

**Additional file 2.** Ethical approval documents and all other ethical bodies that approved our study in the various centers involved.

**Additional file 3.** CRF in Chinese and outline in English.

**Additional file 4.** Code for nomograms.

## Data Availability

The dataset used and analyzed during the current study is available from the corresponding author on reasonable request.

## Abbreviations

AKI: Acute Kidney Injury
AKIN: Acute Kidney Injury Network
APACHE II: Acute Physiology Age and Chronic Health Evaluation II
CI: Confidence interval
CKD: Chronic Kidney Disease
CRF: Case Reported Form
CRRT: Continuous Renal Replacement Therapy
CVVH: Continuous Veno-Venous Hemofiltration
GFR: Glomerular Filtration Rate
HR: Hazard Ratio
ICU: Intensive Care Unit
IHD: Intermittent Hemodialysis
IQR: Interquartile Range
IRRT: Intermittent Renal Replacement Therapy
KDIGO: Kidney Disease Improving Global Outcomes
LOS: Length Of Stay
MDRD: Modification of Diet in Renal Disease
OR: Odds Ratio
RIFLE: Risk, Injury, Failure, Loss, End-stage
RRT: Renal Replacement Therapy
SAPS II: Simplified Acute Physiology Score II
SOFA: Sequential Organ Failure Assessment
WH/WD: Withholding or Withdrawal of Life-sustaining Treatments
