# Supplementary material for: Epidemiology of acute kidney injury in intensive care units in Beijing: the multi-center BAKIT study
Source: BMC Nephrol. 2019 Dec 16;20:468. doi: 10.1186/s12882-019-1660-z (PMC6915890; doi:10.1186/s12882-019-1660-z)
Supplement: Supplementary file 1 — Additional file 1. Full list of participating hospitals. [file 12882_2019_1660_MOESM1_ESM.pdf]

Appendix 1. Full list of participating hospital:

1. Peking University Third Hospital
2. Peking University First Hospital
3. Peking University People's Hospital
4. Beijing Geriatric Hospital
5. Beijing No. 6 Hospital
6. Beijing Shunyi District Hospital, Capital Medical University
7. Beijing Luhe Hospital, Capital Medical University
8. Fuwai Hospital
9. First Hospital Affiliated to the Chinese PLA General Hospital, the 304 Hospital
10. Huaxin Hospital, First Affiliated Hospital of Tsinghua University
11. Anzhen Hospital, Capital Medical University
12. Chaoyang Hospital, Capital Medical University, RICU
13. Chaoyang Hospital, , Capital Medical University, SICU
14. Ditan Hospital, Capital Medical University
15. Shijitan Hospital, Capital Medical University
16. Tiantan Hospital, Capital Medical University
17. Tongren Hospital, Capital Medical University
18. Youyi Hospital, Capital Medical University
19. Youan Hospital, Capital Medical University
20. Fuxing Hospital, Capital Medical University
21. Xuanwu Hospital, Capital Medical University, NICU
22. Xuanwu Hospital, Capital Medical University, SICU
23. Beijing Hospital
24. General Hospital of Armed Police
25. Chinese PLA General Hospital, the 301 Hospital
26. The 309 Chinese PLA Hospital
27. Chinese PLA Naval General Hospital
28. Chinese PLA Air Force General Hospital
29. Peking Union Medical College Hospital
30. China-Japan Friendship Hospital
