# Supplementary material for: Epidemiology of acute kidney injury in intensive care units in Beijing: the multi-center BAKIT study
Source: BMC Nephrol. 2019 Dec 16;20:468. doi: 10.1186/s12882-019-1660-z (PMC6915890; doi:10.1186/s12882-019-1660-z)
Supplement: Supplementary file 2 — Additional file 2. Ethical approval documents and all other ethical bodies that approved our study in the various centers involved. [file 12882_2019_1660_MOESM2_ESM.pdf]

首都医科大学附属复兴医院伦理委员会
Fu Xing Hospital,Capital Medical University IRB

伦 理 审 查 批 件
IRB Review Approval Notice

**批件号** Approval Notice Number	2010FXHEC—KY026		
**临床研究名称** Name of Project	综合 ICU 急性肾损伤的流行病学研究		
**是否基金资助及名称** Project declarer	☑是 □否 **基金名称：首都临床特色应用研究**		
**承担科室** Research department	**重症医学科**		
**研究形式** Responsibility type	☑ **负责** □**参加** Initiator / Participant	**负责单位** Initiator	**首都医科大学附属复兴医院**
**研究者签字** Main researcher	**席修明**	**手机：** Phone No.	18618238611
**联系人** Contact person	**闻英**	**手机：** Phone No.	13683512745
**审查类别** Review type	**初次审查**	**审查方式** Review way	**会议审查**
**审查日期** Review date	**2010.6.25**	**审查地点** Address	**第四会议室**
**审查委员** Cencor	王永利、白　明、刘桂凤、宋　炎、张进生、杜雪平<br>李东霞、骆成玉、杨　明、黄　光、李晓萍		
**审查文件** **Files**	（注明版本号/日期，可另附页）<br>1. 医学伦理审查申请表（2010 年 6 月 20 日）<br>2. 研究方案及病例报告表等相关资料（第 1 版， 2010 年 6 月 1 日）<br>3. 研究人员的简历<br>4. 研究人员名单、职责分工<br>5. 科研项目批文/任务书		

伦理委员会审查意见：
Cencor opinion

1. 研究者的资格与经验：　☑符合要求　□不符合要求；
2. 研究方案及病例报告表：　☑适当，符合科学性和伦理原则　□不适当；
3. 受试者可能遭受的风险程度与研究预期的受益相比：☑适当　□不适当；
4. 向受试者（或其家属、监护人、法定代理人）提供的有关信息资料完整易懂，获得知情同意的方法：　☑适当　□不适当；
5. 参与者隐私的保护：　☑有　□无
6. 研究人员与受试者之间：　☑无利益冲突　□有利益冲突。

根据 SFDA《药物临川该实验伦理审查工作指导原则》（2010）、卫生部《涉及人的生物医学研究伦理审查办法（试行）》（2007）、国家中医药管理局《中医药临床研究伦理审查管理规范》（2010）、SFDA《药物临床试验质量管理规范(2003)》、WMA《赫尔辛基宣言》和 CIOMS《人体生物医学研究国际道德指南》的伦理原则，经本伦理委员会审查：

☑**同意实施**　　□**同意修正后实施**　　□**不同意实施**

由于课题为非干预性临床观察性研究，且所有患者均为匿名，全部信息对于课题负责单位研究人员以外的人员保密，故申请免于知情同意。

其他意见：

请遵循伦理委员会批准的方案开展临床试验/研究，保护受试者的健康与权力。

试验/研究开始前，建议申请人完成临床试验/研究注册。

试验/研究过程中若变更主要研究者，对临床试验/研究方案、知情同意书等的任何修改，请申请人提交修正案审查申请。

发生严重不良事件，请申请人及时提交严重不良事件报告；紧急报告之后，尽快提交详细的严重不良事件随访报告。

请按照伦理委员会规定的期限跟踪审查，申请人在截止日期前1个月提交试验/研究进展报告；当出现任何可能显著影响试验/研究进行、或增加受试者风险的情况时，请申请人立即向伦理委员会提交书面报告。

试验/研究纳入了不符合纳入标准或符合排除标准的受试者，符合中止试验规定而未让受试者退出试验/研究，给予错误治疗或计量，给予方案禁止的合并用药等没有遵从方案开展试验/研究的情况；或可能对受试者的权益/健康、以及试验/研究的科学性造成不良影响等违背 GCP 原则的情况，请申办者/监查员/研究者提交违背方案报告。

申请人暂停或提前终止临床试验/研究，请及时提交暂停/终止试验/研究报告。

完成临床试验/研究，请申请人提交结题报告。

本项临床试验/研究应当在批准之日起一年内实施，逾期未实施的，本批件自行废止。

**跟踪审查频率**<br>Regular tracking review frequency	**请于 2011 年 12 月 31 日 前 1 个月提交进展报告**
**有效期**<br>Validity period	**2010 年 7 月 1 日 — 2012 年 12 月 31 日**
**主任委员签字/盖章**<br>Commissioner signature	罗斐
**伦理委员会盖章**<br>IRB signature	首都医科大学附属复兴医院医学伦理委员会
**签发日期**<br>Date	**2010 年 6 月 27 日**

All other ethical bodies that approved our study in the various centers involved:

The institutional review board of Peking Union Medical College Hospital

The institutional review board of Beijing Tongren Hospital, Capital Medical University

The institutional review board of Beijing Tiantan Hospital affiliated to Capital Medical University

The institutional review board of Beijing Friendship Hospital, Capital Medical University

The institutional review board of Beijing Ditan Hospital , Capital Medical University

The institutional review board of Beijing Chaoyang Hospital, Capital Medical University

The institutional review board of Xuanwu Hospital, Capital Medical University

The institutional review board of Peking University Third Hospital

The institutional review board of Peking University First Hospital

The institutional review board of Peking University People's Hospital

The institutional review board of China-Japan Friendship Hospital

The institutional review board of The 309th Hospital of Chinese People's Liberation Army

The institutional review board of Beijing Shijitan Hospital, Capital Medical University

The institutional review board of Fuwai Hospital, China Academy of Medical Science and Peking Union Medical College

The institutional review board of Air Force General Hospital of Chinese People's Liberation Army

The institutional review board of The First Affiliated Hospital of General Hospital of People's Liberation Army

The institutional review board of Navy General Hospital

The institutional review board of The Luhe Teaching Hospital of the Capital Medical University

The institutional review board of Beijing Anzhen Hospital, Capital Medical University

The institutional review board of Beijing Hospital

The institutional review board of General Hospital of Armed Police Forces

The institutional review board of The General Hospital of People's Liberation Army

The institutional review board of Beijing YouAn Hospital, Capital Medical University

The institutional review board of HuaXin Hospital, First Hospital of Tsinghua University

The institutional review board of Beijing Shunyi Hospital of China Medical University

The institutional review board of Beijing Geriatric Hospital

The institutional review board of Beijing No.6 Hospital
