# Supplementary material for: Epidemiology of acute kidney injury in intensive care units in Beijing: the multi-center BAKIT study
Source: BMC Nephrol. 2019 Dec 16;20:468. doi: 10.1186/s12882-019-1660-z (PMC6915890; doi:10.1186/s12882-019-1660-z)
Supplement: Supplementary file 3 — Additional file 3. CRF in Chinese and outline in English. [file 12882_2019_1660_MOESM3_ESM.pdf]

CRF in Chinese and outline in English

表 1 人口学资料、诊断及伴随疾病

1 人口学资料:

1.1 出生日期: □□□□/□□/□□(年/月/日)

1.2 性别: □男 □女

1.3 民族: □汉 □其他（请注明）

1.4 身高：□□□cm

1.5 体重：□□□kg

1.6 医疗费用种类：□医疗保险 □公费医疗 □新农合 □医疗保险（一老一小） □自费 □其他（请注明）__________

2 入院及入 ICU 情况

2.1 入院时间：□□□□/□□/□□ (年/月/日)

2.2 主要入院诊断：

2.3 入 ICU 时间：□□□□/□□/□□:□□(年/月/日/24 小时制时间)

2.4 转入 ICU 情况: □计划内转入（至少提前 12 小时）
□非计划内转入

2.5 转入科室：□急诊 □病房 □术后转入（以术后监护治疗为目的） □本院其他 ICU □外院

2.6 入 ICU 主要原因及并发症（可多选）:

□手术后治疗及监护 □呼吸系统疾病
□心跳/呼吸骤停后 □消化系统疾病
□心血管系统疾病 □中枢神经系统疾病

Page 1: data of demographics, admission time point, source of patients (i.e. emergency department, general ward, other kind of ICU), reasons for admission

□中毒或药物过量　　　　□创伤

□内分泌和代谢性疾病

□其他（请注明）

3　入 ICU 时主要脏器损害（可多选）

□呼吸衰竭　　　　□梗阻性休克

□心源性休克　　　　□DIC

□低容量休克　　　　□急性肝衰竭

□感染性休克

□其他（请注明）

4　入 ICU 时手术情况：□未手术 □择期手术　□急诊手术

4.1　手术种类

□心脏手术　　　　□消化道手术

□胸部手术　　　　□肾脏手术

□脊椎及脊髓手术　　　　□其他腹部手术

□四肢手术　　　　□颅脑及神经系统手术

□移植手术（□肝脏 □心脏 □骨髓 □胰腺）

□其他手术（请注明）

5　基础血肌酐：□□□.□μmol/L 或□□.□mg/dl（患者入 ICU 前 3 月内最低血肌酐值，若入 ICU 前 3 个月未做过化验，可参照工作手册中的表格查找基础血肌酐参考值。

6　伴随情况（可多选）

□恶性肿瘤　　　　□血液系统恶性肿瘤

Page 2: baseline serum creatinine, co-morbidity, organ failure

□转移癌　　□高血压

□心功能 IV 级　　□COPD

□慢性肝脏病　　□冠心病

□艾滋病　　□免疫功能损害

□慢性肾脏病（伴肾功能不全）

□慢性肾脏病（不伴肾功能不全）

□糖尿病 □胰岛素治疗□非胰岛素治疗

7 入 ICU 前使用以下药物（可多选）□是 □否

7.1 2 周内曾经使用 □是 □否

□氨基糖甙类抗生素　　□两性霉素 B

□糖肽类抗生素　　□甘露醇

□造影剂

□血管活性药物（不含扩血管类）

7.2 3 个月内曾经使用 □是 □否

□非甾体抗炎药（NSAID） □ACE-I/ARB □他汀类降脂药

8 患者家属联系方式（如有）____________

Page 3: medications administrated before ICU admission

表 2　入 ICU 首个 24 小时

1　生命体征

1.1　心率[①③]：最低值□□□次/分　最高值□□□次/分

1.2　收缩压[③]：最低值□□□mmHg 或□□.□kPa

1.3　平均动脉压[①②]：最低值□□□mmHg 或□□.□kPa

最高值□□□mmHg 或□□.□kPa

1.4　体温[①③]：最低值□□.□℃　最高值□□.□℃

1.5　呼吸频率[①]：最低值□□次/分　最高值□□次/分

2　实验室检查

2.1　血气分析（同一次）

2.1.1　pH 值[①]（最低值）：□.□□□

2.1.2　$PaCO_2$[①]：□□□mmHg

2.1.3　PaO_2[①②③]：□□□mmHg

2.1.4　HCO_3^-[③]：□□.□mmol/L

2.2　血常规

2.2.1　白细胞[①③]：最低值□□.□×10^9 /L最高值□□.□×10^9 /L

2.2.2　红细胞压积[①]：最低值□□.□%

2.2.3　血小板[②]：最低值□□□×10^9 /L最高值□□□×10^9 /L

2.3　血生化肝肾功

2.3.1　血清钾[①③]：最低值□.□□mmol/L

最高值□.□□mmol/L

2.3.2　血清钠[①]：最低值□□□.□mmol/L

Page 4: vital signs, laboratory tests

最高值□□□.□mmol/L

2.3.3 尿素氮[③]：最高值□□.□mmol/L 或□.□□mg/dl

2.3.4 肌酐[①②]：最高值□□□.□μmol/L 或□□.□mg/dl

2.3.5 总胆红素[②③]：最高值□□□.□μmol/L 或□□.□mg/dl

2.4 尿量[②③]（请填写在表 3 的尿量记录中，参照护理记录按相应时间点填写）

2.5 GCS 评分[②③]：□□分

2.6 机械通气[③]（有创）：□是　□否

2.7 吸入氧浓度(FiO_2)[②③] 0. □□（**以小数计**，非机械通气时参照工作手册选择）

2.8 应用血管活性药物[②]：□是　□否

2.8.1 多巴胺（最高剂量）：□无　□≤5μg/kg/min
□＞5 且≤15μg/kg/min　□＞15μg/kg/min

2.8.2 肾上腺素（最高剂量）：□无　□≤0.1μg/kg/min
□＞0.1μg/kg/min

2.8.3 去甲肾上腺素（最高剂量）：□无　□ ≤0.1μg/kg/min
□＞0.1μg/kg/min

2.8.4 使用多巴酚丁胺：□是　□否

3 已打分者可**直接填写分值**，并尽可能提供原始打分表。未打的分不用自行打，请**填写标注该分数编号的项目即可**。

① APACHE II______　②SOFA______　③SAPS II______

Page 5: treatment including mechanical ventilation and vasopressors, GCS, APACHE II, SAPS II, and SOFA score

表 3 ICU 每日数据

入 ICU 天数		1	2	3	4	5	6	7	8	9	10
日期		月日	月日	月日	月日	月日	月日	月日	月日	月日	月日
肌酐最高值	μmol/L										
	mg/dL										
24 小时液体平衡（ml）											
行 RRT（肾脏替代治疗）		□是<br>□否	□是<br>□否	□是<br>□否	□是<br>□否	□是<br>□否	□是<br>□否	□是<br>□否	□是<br>□否	□是<br>□否	□是<br>□否
PaO_2/FiO_2 mmHg											
机械通气		□是<br>□否	□是<br>□否	□是<br>□否	□是<br>□否	□是<br>□否	□是<br>□否	□是<br>□否	□是<br>□否	□是<br>□否	□是<br>□否
血小板 $\times10^9/L$	最低值										
	最高值										
总胆红素	μmol/L										
	mg/dL										
平均动脉压 mmHg/kPa	最低值										
	最高值										
GCS 评分											

Page 6: daily information of serum creatinine, fluid balance, treatment including mechanical ventilation and RRT, other laboratory results, vital signs, GCS score

入 ICU 天数	1	2	3	4	5	6	7	8	9	10
当日多巴胺最高剂量<br>μg/kg/min	□无<br>□ ≤5<br>□>5<br>且≤15<br>□>15	□无<br>□ ≤5<br>□>5<br>且≤15<br>□>15	□无<br>□ ≤5<br>□>5<br>且≤15<br>□>15	□无<br>□ ≤5<br>□>5<br>且≤15<br>□>15	□无<br>□ ≤5<br>□>5<br>且≤15<br>□>15	□无<br>□ ≤5<br>□>5<br>且≤15<br>□>15	□无<br>□ ≤5<br>□>5<br>且≤15<br>□>15	□无<br>□ ≤5<br>□>5<br>且≤15<br>□>15	□无<br>□ ≤5<br>□>5<br>且≤15<br>□>15	□无<br>□ ≤5<br>□>5<br>且≤15<br>□>15
当日肾上腺素最高剂量<br>μg/kg/min	□无<br>□≤0.1<br>□>0.1	□无<br>□≤0.1<br>□>0.1	□无<br>□≤0.1<br>□>0.1	□无<br>□≤0.1<br>□>0.1	□无<br>□≤0.1<br>□>0.1	□无<br>□≤0.1<br>□>0.1	□无<br>□≤0.1<br>□>0.1	□无<br>□≤0.1<br>□>0.1	□无<br>□≤0.1<br>□>0.1	□无<br>□≤0.1<br>□>0.1
当日去甲肾上腺素最高剂量<br>μg/kg/min	□无<br>□≤0.1<br>□>0.1	□无<br>□≤0.1<br>□>0.1	□无<br>□≤0.1<br>□>0.1	□无<br>□≤0.1<br>□>0.1	□无<br>□≤0.1<br>□>0.1	□无<br>□≤0.1<br>□>0.1	□无<br>□≤0.1<br>□>0.1	□无<br>□≤0.1<br>□>0.1	□无<br>□≤0.1<br>□>0.1	□无<br>□≤0.1<br>□>0.1
应用多巴酚丁胺	□是<br>□否	□是<br>□否	□是<br>□否	□是<br>□否	□是<br>□否	□是<br>□否	□是<br>□否	□是<br>□否	□是<br>□否	□是<br>□否
以下利尿剂应用<br>1 呋塞米<br>2 托拉塞米<br>3 丁尿胺	□无<br>□1<br>□2<br>□3<br>□其他<br>（请注明）	□无<br>□1<br>□2<br>□3<br>□其他<br>（请注明）	□无<br>□1<br>□2<br>□3<br>□其他<br>（请注明）	□无<br>□1<br>□2<br>□3<br>□其他<br>（请注明）	□无<br>□1<br>□2<br>□3<br>□其他<br>（请注明）	□无<br>□1<br>□2<br>□3<br>□其他<br>（请注明）	□无<br>□1<br>□2<br>□3<br>□其他<br>（请注明）	□无<br>□1<br>□2<br>□3<br>□其他<br>（请注明）	□无<br>□1<br>□2<br>□3<br>□其他<br>（请注明）	□无<br>□1<br>□2<br>□3<br>□其他<br>（请注明）

Page 7: daily information of medications including vasopressors and diuretics

ICU 每日数据——尿量（ml）

时间 / 入 ICU 天数	8 am	9 am	10 am	11 am	12 n	13 pm	14 pm	15 pm	16 pm	17 pm	18 pm	19 pm	20 pm	21 pm	22 pm	23 pm	24 pm	1 am	2 am	3 am	4 am	5 am	6 am	7 am
**1**																								
**2**																								
**3**																								
**4**																								
**5**																								
**6**																								
**7**																								
**8**																								
**9**																								
**10**																								

几点记录的尿量就填写在几点，请在时间表中标注"↑"表示转入 ICU，标注"↓"表示转出或死亡。

Page 8: daily urine output

表 4 AKI

1. AKI 诱因（可多选）

☐应用肾毒性药物（诊断 AKI 前 72 小时内）

☐氨基糖甙类抗生素	☐脓毒症（sepsis）
☐糖肽类抗生素	☐低血容量
☐两性霉素 B	☐低心排
☐造影剂	☐肝肾综合征
☐非甾体抗炎药物(NSAID's)	☐肾后性梗阻
☐ACEI/ARB	
☐其他（请注明）	

3. 本次住 ICU 肾功能最差分级：☐无 AKI ☐R ☐I ☐F

Page 9: possible risk factors of AKI, diagnosis and stage of AKI

表5 肾脏替代治疗（RRT）

1. 肾脏替代治疗（RRT）开始日期：□□□□/□□/□□（年/月/日）
2. 开始肾脏替代治疗（RRT）原因（可多选）

□无尿/少尿　　□严重代谢性酸中毒

□高钾血症　　□严重脏器水肿

□高钠/低钠血症　　□药物过量

□其他（请说明）________________

3. 穿刺管路种类：□双腔血滤管　　□三腔血滤管
4. 穿刺部位

□左颈内静脉　　□右颈内静脉

□左股静脉　　□右股静脉

□左锁骨下静脉　　□右锁骨下静脉

□A-V瘘　　□其他（请注明）

5 初始治疗模式

□CVVH　　□IHD

□CVVHD　　□SCUF

□CVVHDF　　□SLED

□HVHF　　□其他（请注明）__________

6 主要治疗模式（最长时间）

□CVVH　　□IHD

□CVVHD　　□SCUF

□CVVHDF　　□SLED

Page 10: detailed information of RRT including date of initiating RRT, reasons to start RRT, , catheter insertion sites, mode of RRT

☐HVHF ☐其他（请注明）________

7 初始抗凝方式

☐无抗凝 ☐低分子肝素

☐普通肝素 ☐枸橼酸钠

☐普通肝素+鱼精蛋白 ☐其他（请注明）________

8 主要抗凝方式（最长时间）

☐无抗凝 ☐低分子肝素

☐普通肝素 ☐枸橼酸钠

☐普通肝素+鱼精蛋白 ☐其他（请注明）________

9 置换液输入方式（CVVH 及 CVVHDF 时填写）

☐前稀释 ☐后稀释 ☐前稀释+后稀释

10 出血 ☐有 ☐无

10.1 出血部位（可多选）

☐穿刺部位出血或渗血 ☐颅内出血

☐皮肤瘀紫或瘀斑 ☐气道出血

☐消化道出血 ☐其他部位（请注明）______

10.2 出血与抗凝剂相关性

☐无关 ☐有关 ☐可能有关

Page 11: anticoagulation pattern, adverse events

表 6 全身性感染或脓毒症（Sepsis）

1. Sepsis（诊断标准见工作手册）发生时间

   □从未发生　　□入 ICU 时　□入 ICU 后

2. sepsis 部位

   □呼吸系统　　□泌尿系统
   □胃肠道　　□皮肤软组织
   □腹腔　　□神经系统
   □血流感染　　□其他（请注明）________

3 院内获得性感染（感染发生在入院>48h 后）：□是　□否

4 sepsis 与 AKI 发生：□无关　□有关　□可能有关

Page 12: co-morbidity of sepsis

表 7 预后

1 决定撤销生命支持治疗：□是 □否

1.1 决定撤销生命支持治疗时间：□□□□/□□/□□(年/月/日)

2 ICU 预后及参数

2.1 转出 ICU 时存活：□是 □否

2.2 转出 ICU/死亡时间：□□□□/□□/□□(年/月/日)

2.3 转出 ICU 血肌酐（离转出 ICU 最近时间点的血肌酐）：

□□□.□μmol/L 或□□.□mg/dL

2.4 转出 ICU 前 24 小时尿量：□□□□ml/24h

2.5 转出 ICU 时仍需要肾脏替代治疗：□是 □否

2.5.1肾脏替代治疗方式

□IHD □PD □其他（请注明）

3 28 天预后

3.1 入 ICU 后第 28 天存活：□是 □否

3.2 第 28 天血肌酐（离第 28 天最近时间点的血肌酐）：

□□□.□μmol/L 或□□.□mg/dL

3.3 第 28 天仍需要肾脏替代治疗：□是 □否

3.3.1肾脏替代治疗方式 ：

□CVVH □SLED

□CVVHD □IHD

□CVVHDF □PD

□SCUF □其他（请注明）________

Page 13: outcomes including withdrawal of life-sustaining treatment, 28-day mortality, kidney discovery on ICU discharge

4 住院预后

4.1 出院时存活:□是 □否

4.2 出院/死亡时间：□□□□/□□/□□(年/月/日)

4.3 出院时血肌酐（离出院最近时间点肌酐）：

□□□.□μmol/L 或□□.□mg/dL

4.4 出院时仍需要肾脏替代治疗：□是 □否

4.4.1 肾脏替代治疗方式 ：

□IHD □PD □其他（请注明）________

5 ICU 住院总费用：□□□. □(万元)

Page 14: hospital mortality, kidney discovery on hospital discharge
