# Supplementary material for: Epidemiology of acute kidney injury in intensive care units in Beijing: the multi-center BAKIT study
Source: BMC Nephrol. 2019 Dec 16;20:468. doi: 10.1186/s12882-019-1660-z (PMC6915890; doi:10.1186/s12882-019-1660-z)
Supplement: Supplementary file 4 — Additional file 4. Code for nomograms. [file 12882_2019_1660_MOESM4_ESM.pdf]

```
#nomogram of logistic regression
library(rms)
read.table("/Users/zhuyibing/Desktop/risk-zhangqi.csv",header=TRUE,sep=";")->data #181204修改
ddist<-datadist(data)
options(datadist="ddist")
model<-lrm(AKI~Cr+APACHE+SOFA+Sepsis+Drugs,data)
model
nom<-nomogram(model,
        lp.at=seq(-2,4,by=0.5),
        fun=function(x)1/(1+exp(-x)),
        funlabel="Risk of AKI",
        fun.at=c(0.05,seq(0.1,0.9,by=0.1),0.95),
        conf.int=c(0.1,0.3))
nom
plot(nom,lplabel="linear Predictor",
fun.side=c(3,1,1,1,3,1,3,1,1,1,3),
label.every=2,
col.conf=c("red","green"),
conf.space=c(0.1,0.3),
col.grid=gray(c(0.8,0.95)))
#Results:
#Logistic Regression Model
#
#lrm(formula = AKI ~ Cr + APACHE + SOFA + Sepsis + Drugs, data = data)
#
#                 Model Likelihood     Discrimination    Rank Discrim.
#                    Ratio Test          Indexes           Indexes
#Obs         3107    LR chi2     628.32    R2       0.244    C       0.744
#0          1523    d.f.            5    g        1.201    Dxy     0.489
#1          1584    Pr(> chi2) <0.0001    gr       3.322    gamma   0.489
#max |deriv| 2e-05                        gp       0.246    tau-a   0.244
#                                      Brier    0.204
#         Coef    S.E.   Wald Z Pr(>|Z|)
#Intercept -2.3092 0.1363 -16.95 <0.0001
#Cr         0.0043 0.0010   4.26 <0.0001
#APACHE     0.0531 0.0070   7.56 <0.0001
#SOFA       0.1471 0.0146  10.06 <0.0001
#Sepsis     0.6311 0.0967   6.53 <0.0001
#Drugs      0.3411 0.0859   3.97 <0.0001
exp(0.0043) #Cr
exp(0.0531) #APACHE
exp(0.1471) #SOFA
exp(0.6311) #SEPSIS
exp(0.3411) #DRUG
exp(0.0043-1.96*0.0010) #Cr-
exp(0.0043+1.96*0.0010) #Cr+
exp(0.0531-1.96*0.0070) #APACHE-
exp(0.0531+1.96*0.0070) #APACHE+
exp(0.1471-1.96*0.0146) #SOFA-
exp(0.1471+1.96*0.0146) #SOFA+
exp(0.6311-1.96*0.0967) #SEPSIS-
exp(0.6311+1.96*0.0967) #SEPSIS+
exp(0.3411-1.96*0.0859) #DRUG-
exp(0.3411+1.96*0.0859) #DURG+
#Results
```

```
#> exp(0.0043)
#[1] 1.004309
#> exp(0.0531)
#[1] 1.054535
#> exp(0.1471)
#[1] 1.15847
#> exp(0.6311)
#[1] 1.879677
#> exp(0.3411)
#[1] 1.406494
#> exp(0.0043-1.96*0.0010) #Cr-
#[1] 1.002343
#> exp(0.0043+1.96*0.0010) #Cr+
#[1] 1.00628
# > exp(0.0531-1.96*0.0070) #APACHE-
#[1] 1.040166
#> exp(0.0531+1.96*0.0070) #APACHE+
#[1] 1.069103
#> exp(0.1471-1.96*0.0146) #SOFA-
#[1] 1.125789
#> exp(0.1471+1.96*0.0146) #SOFA+
#[1] 1.192099
#> exp(0.6311-1.96*0.0967) #SEPSIS-
#[1] 1.555144
#> exp(0.6311+1.96*0.0967) #SEPSIS+
#[1] 2.271935
#> exp(0.3411-1.96*0.0859) #DRUG-
#[1] 1.188552
#> exp(0.3411+1.96*0.0859) #DURG+
#[1] 1.664399

#nomogram of Cox regression
library(survival)
read.table("/Users/zhuyibing/Desktop/181219cox.csv",header=TRUE,sep=";")->a #AKI
time<-a[,1]
status<-a[,2]
KDIGO<-a[,3]
Sepsis<-a[,4]
age<-a[,5]
APACHE<-a[,6]
SOFA<-a[,7]
model<-cph(Surv(time,status)~KDIGO+Sepsis+age+APACHE+SOFA,a,surv=TRUE)
model
ddist<-datadist(a)
options(datadist="ddist")
surv<-Survival(model)
nom<-nomogram(model,
            fun=list(function(x) surv(28,x)),
            funlabel=c("28-day sur.prob"),
            lp=F)
plot(nom,
    fun.side=list(c(rep(1,7),3,1,3),c(rep(1,7),3,1,3)),
    col.grid=c("red","green"))

#Results
#Cox Proportional Hazards Model
```

```
#cph(formula = Surv(time, status) ~ KDIGO + Sepsis + age + APACHE +
#   SOFA, data = a, surv = TRUE)
#
#                  Model Tests       Discrimination
#                                    Indexes
#Obs      3107    LR chi2    364.22    R2       0.118
#Events    543    d.f.            5    Dxy      0.433
#Center 2.0259    Pr(> chi2) 0.0000    g        0.900
#                 Score chi2 423.35    gr       2.460
#                  Pr(> chi2) 0.0000
#
#        Coef   S.E.   Wald Z Pr(>|Z|)
#KDIGO  0.3020 0.0420 7.19   <0.0001
#Sepsis 0.4908 0.0980 5.01   <0.0001
#age    0.0145 0.0029 5.09   <0.0001
#APACHE 0.0341 0.0072 4.71   <0.0001
#SOFA   0.0268 0.0151 1.77   0.0764
exp(0.3020) #KDIGO
exp(0.4908) #SEPSIS
exp(0.0145) #AGE
exp(0.0341) #APACHE
exp(0.0268) #SOFA
exp(0.3020-1.96*0.0420) #KDIGO-
exp(0.3020+1.96*0.0420) #KDIGO+
exp(0.4908-1.96*0.0980) #SEPSIS-
exp(0.4908+1.96*0.0980) #SEPSIS+
exp(0.0145-1.96*0.0029) #AGE-
exp(0.0145+1.96*0.0029) #AGE+
exp(0.0341-1.96*0.0072) #APACHE-
exp(0.0341+1.96*0.0072) #APACHE+
exp(0.0268-1.96*0.0151) #SOFA-
exp(0.0268+1.96*0.0151) #SOFA+
#Results
#> exp(0.3020) #KDIGO
#[1] 1.352561
#> exp(0.4908) #SEPSIS
#[1] 1.633623
#> exp(0.0145) #AGE
#[1] 1.014606
#> exp(0.0341) #APACHE
#[1] 1.034688
#> exp(0.0268) #SOFA
#[1] 1.027162
#> exp(0.3020-1.96*0.0420) #KDIGO-
#[1] 1.245678
#> exp(0.3020+1.96*0.0420) #KDIGO+
#[1] 1.468615
#> exp(0.4908-1.96*0.0980) #SEPSIS-
#[1] 1.348132
#> exp(0.4908+1.96*0.0980) #SEPSIS+
#[1] 1.979571
#> exp(0.0145-1.96*0.0029) #AGE-
#[1] 1.008855
#> exp(0.0145+1.96*0.0029) #AGE+
#[1] 1.020389
```

```
#> exp(0.0341-1.96*0.0072) #APACHE-
#[1] 1.020189
#> exp(0.0341+1.96*0.0072) #APACHE+
#[1] 1.049393
#> exp(0.0268-1.96*0.0151) #SOFA-
#[1] 0.9972079
#> exp(0.0268+1.96*0.0151) #SOFA+
#[1] 1.058017
```
